# Brain-correlates of processing local dependencies within a statistical learning paradigm

**DOI:** 10.1038/s41598-022-19203-7

**Published:** 2022-09-12

**Authors:** Vera Tsogli, Stavros Skouras, Stefan Koelsch

**Affiliations:** Department for Biological and Medical Psychology, University of Bergen, Postboks 7807, 5020 Bergen, Norway

**Keywords:** Psychology, Cognitive neuroscience, Auditory system

## Abstract

Statistical learning refers to the implicit mechanism of extracting regularities in our environment. Numerous studies have investigated the neural basis of statistical learning. However, how the brain responds to *violations* of auditory regularities based on prior (implicit) learning requires further investigation. Here, we used functional magnetic resonance imaging (fMRI) to investigate the neural correlates of processing events that are irregular based on learned local dependencies. A stream of consecutive sound triplets was presented. Unbeknown to the subjects, triplets were either (a) standard, namely triplets ending with a high probability sound or, (b) statistical deviants, namely triplets ending with a low probability sound. Participants (n = 33) underwent a learning phase outside the scanner followed by an fMRI session. Processing of statistical deviants activated a set of regions encompassing the superior temporal gyrus bilaterally, the right deep frontal operculum including lateral orbitofrontal cortex, and the right premotor cortex. Our results demonstrate that the violation of local dependencies within a statistical learning paradigm does not only engage sensory processes, but is instead reminiscent of the activation pattern during the processing of local syntactic structures in music and language, reflecting the online adaptations required for predictive coding in the context of statistical learning.

## Introduction

Our external world and our life in general, are far from a random continuum of events, and rather contain a certain degree of structure and regularity^1^. Humans are endowed with the capability to detect regularities and form predictions about future events. In recent years, statistical learning has been suggested as a key mechanism for the detection of regularities (for reviews see Refs.^2–4^). The crucial role of prediction in perception, cognition and action has been well established^5,6^, but recent accounts have also identified the central role of prediction in statistical learning^7–9^. In this perspective, a fundamental question that arises is how the brain responds to violations of predictions that result from implicit statistical learning. Implicit adaptation to regularities has been mainly studied from two research traditions, implicit learning and statistical learning which employ different experimental paradigms but as it has been argued they study the same underlying mechanism^10,11^. Both research paradigms have investigated the neural underpinnings of processing structured stimuli, mainly within the context of language acquisition in respect to grammar-rules learning or word segmentation. Although these paradigms have answered important questions regarding the underlying mechanisms of implicit statistical learning, the evidence is scarce about the way the brain responds to unexpected events, occurring in the ongoing stimuli stream and violate the implicitly learned regularities. Non-fMRI studies^12,13^ have examined the impact of prior implicit knowledge on processing unpredictable events. On the other hand, responses to unexpected events have been the main scope of oddball studies using the classical mismatch response known as mismatch negativity (MMN^14^; for a review see Ref.^15^). Nevertheless, these studies did not focus on the brain responses to deviant events within the context of statistical learning. Our research question taps on both mechanisms of implicit statistical learning and deviance detection within the same paradigm and thus differs from previous studies in both domains.

Both implicit and explicit learning research have used the artificial grammar learning paradigm to examine the neural mechanisms of processing stimuli structures with either local (e.g., “The boy was tall.”;^16–23^) or non-local dependencies (e.g., “The boy [that the girl kissed] was tall.”;^20,21,23–26^). In the present study we used local dependencies, yet our research question went beyond the investigation of how the brain processes local dependencies. The focus of our study was rather on the brain responses to deviant local dependencies occurring among standard ones, that as a consequence violate predictions. Previous neuroimaging studies have shown that processing or violations of local dependencies activate the deep frontal and superior temporal areas^20,21^ whereas others report also activation of Broca’s area^16–19^. Artificial grammar learning studies have examined the brain correlates to violations of grammatical dependencies, but these were used as test items for classification purposes during a testing session. Thus, it remains to be investigated how the brain responds to deviant local dependencies embedded in a stream of standard ones within a statistical learning paradigm.

Studies within the domain of statistical learning—to the best of our knowledge—have neither investigated brain responses to breaches of predictions that are based on learned statistical regularities. Instead, the main focus of these studies has been the recognition of word-boundaries within a pause-free stream of concatenated artificial words, a phenomenon known as word segmentation^27^. The main findings from statistical learning fMRI studies are that word learning and recognition are supported by the superior temporal gyrus for both auditory^28–32^ and visual^33^ stimuli, occasionally the inferior frontal gyrus^28–30,32–34^ and the basal ganglia^28,30,35^. The role of the inferior frontal gyrus in statistical learning has also been demonstrated from an source localisation electroencephalographic study^36^. These studies have manipulated the transitional probabilities among the words in order to induce word-boundaries, on the basis that a low transitional probability is indicative of a word-boundary. Nevertheless, statistical learning fMRI studies have not manipulated the transitional probabilities within the words (e.g.: “pretty baby” vs “pretty babies”). The current paradigm aimed to address this gap with a variant of the traditional statistical learning paradigm where transitional probability would vary between and within the sound triplets, and thus examine brain responses to unexpected low-probability events occurring among expected high-probability events (based on prior implicit learning). A similar learning paradigm has been employed to investigate visual statistical learning using event-related brain potentials^37,38^.

How the brain responds to unexpected events, a process known also as deviance detection, has been traditionally investigated in oddball studies using the classical MMN response. Previous neuroimaging oddball studies have shown that unpredicted events activate a cortical network involving the superior temporal gyrus (STG) and occasionally the inferior frontal gyrus (IFG), notably similar to the one observed during statistical learning^39–46^. It is important to note that perception of a deviant event requires first the establishment of a memory representation of the regular event. In a sensory deviance detection paradigm (such as a typical MMN paradigm with a physical deviance, e.g. a pitch deviance) the establishment of a memory representation regarding the standard stimuli requires a few seconds whereas, in a statistical learning paradigm, it requires a longer exposure to the stimuli stream. Thus, in oddball studies, the predictive processes entail regularities that are established on a moment-to-moment basis and represent information accumulated in a timescale of seconds, and in that respect, they are different from regularities established during a statistical learning paradigm. On that basis, in the current statistical learning paradigm, the acquisition of regularities differs because it necessitates longer learning periods, and within this context the brain-correlates of deviance detection are examined.

**Figure 1:**
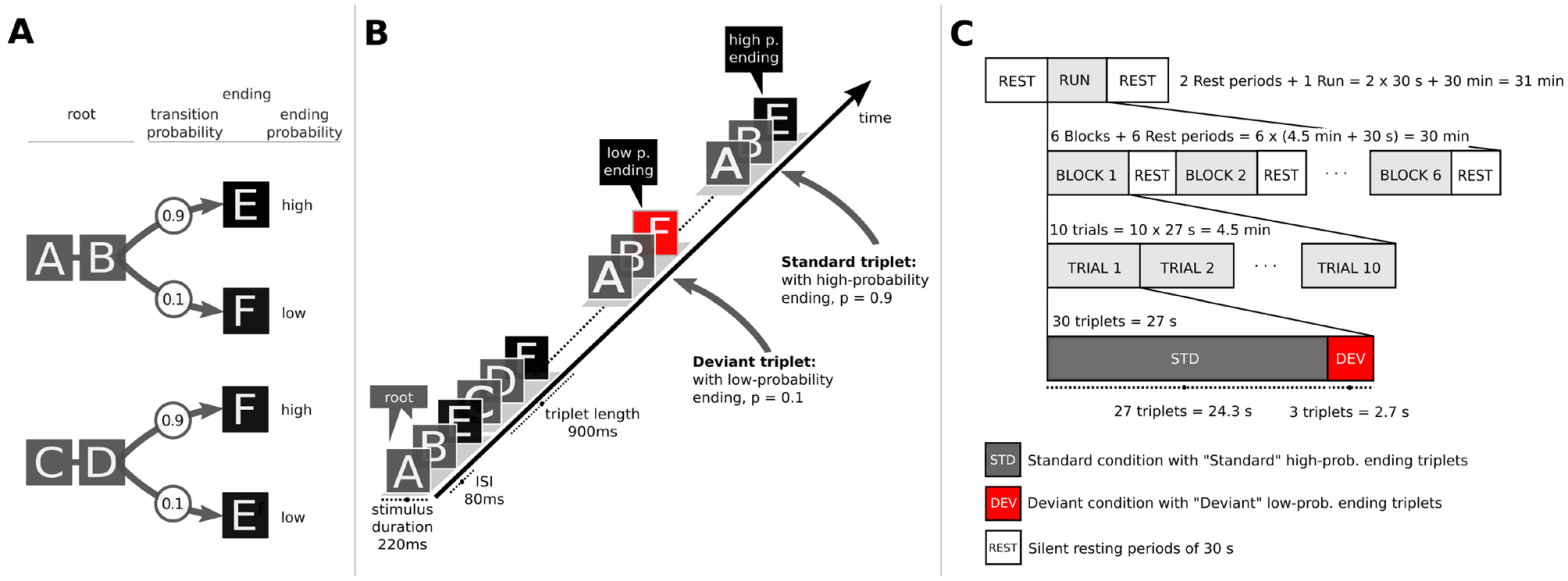
The experimental paradigm and auditory stream inside and outside the scanner. (**A**) The four triplets generated from the 6 sounds. The letters A to E are used to refer to the sounds. The first two items of the triplet form the root (AB and CD) and the last item the triplet ending (E or F). Statistical deviants were created by varying the transitional probability from root to ending within two levels, high (p = 0.9) and low (p = 0.1). Triplet roots (AB or CD) were occurring with a constant transitional probability (p = 0.5) from all of the triplet endings (E or F). (**B**) The auditory stream throughout the learning phase outside the scanner. The triplets were pseudorandomly concatenated and were either “Standard triplets” with high-probability endings (p = 0.9) or “Deviant triplets” with low-probability endings (p = 0.1). (**C**) The auditory stream inside the scanner. Scanning comprised of a single run of approximately 31 min in duration during which 6 blocks of 4.5 min duration each, interleaved with resting periods of 30 sec were presented. Within each block, 10 trials of 27 sec duration each were concatenated. In each trial, 27 consecutive standard triplets were presented followed by 3 deviant triplets.

Other than many previous implicit statistical learning studies, we approached the topic with a relatively new method, namely devising a paradigm that is a fusion of implicit statistical learning and deviance detection paradigms (see Fig. 1) and it was developed based on a previous study^47^. Stimuli were arranged in triplets, in which the transitional probability to the last element was either high (p = 0.9) or low (p = 0.1). High-probability ending triplets were referred as “Standards” whereas low-probability ending triplets were referred to as “Deviants”. We defined three hypotheses. First, beyond the auditory cortex, mismatch responses reflecting the detection of auditory deviance would activate areas associated with violation of local dependencies^16–22^, specifically the temporal and the inferior frontal cortex mediated by parts in the premotor cortex. The scope of the study was to induce implicit statistical learning both outside and inside the scanner and thus our second hypothesis was that the activation pattern would evolve over the duration of a session, revealing the time-course of statistical learning effects. In other words we hypothesised that participants would be still learning inside the scanner. Third, the engaged cortical network would differ between “good” and “bad” learners as ranked by their performance on the behavioural task.

## Results

### Behavioural data outside and inside the scanner

#### Familiarity test (outside the scanner)

At the end of each exposition block (before the fMRI session), a familiarity test was presented to test whether participants had learned the underlying regularities of the stimuli. It was expected that participants would classify the triplets with high probability endings (“Standard” triplets) as more familiar compared with those with low probability endings (“Deviant” triplets; see “Methods”). Participants achieved an mean score of 66.5% (*SEM* = 2.5%) in classifying the standard triplets as more familiar (performance differed significantly from chance level, *p* < .0001). During the debriefing after the fMRI session, participants were asked whether they detected any patterns in the stimuli. Several participants answered affirmatively, however only one (out of 33) was able to describe the triplet structure of the presented stimuli and she performed well above chance level (80.5% correct responses).

#### Cover task (outside and inside the scanner)

Participants were not informed about the statistical regularities underlying the stimuli, but instead were provided with a cover task, namely to detect a (higher-pitched) target sound. During the exposition phase (outside the scanner) participants detected on average 99.4% of the (higher-pitched) target sounds whereas inside the scanner they detected 100%. Thus, participants attended the sounds while they were watching the silent movie.

### Neuroimaging data

**Figure 2:**
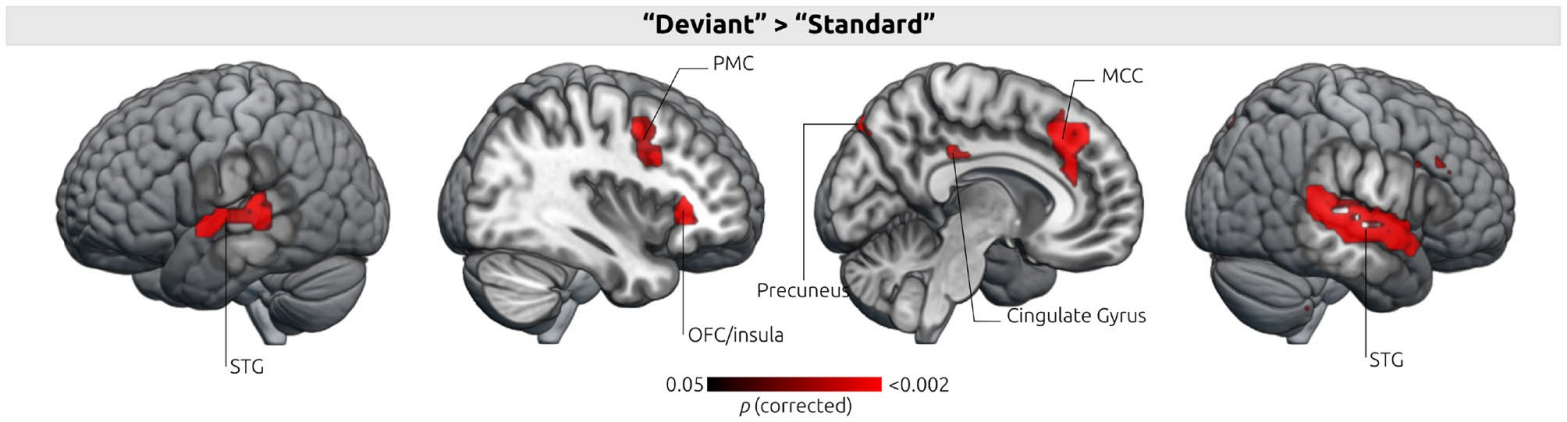
Brain activation pattern during violation of prediction (deviance detection). Activations during presentation of “Deviant” triplets contrasted with activations during presentation of “Standard” triplets (Deviant > Standard). Activation map thresholded at *p* < 0.05 (FDR). Superior Temporal Gyrus (STG); Orbitofrontal cortex (OFC); Premotor Cortex (PMC); Middle Cingulate gyrus (MCC). The figure was created using MRIcroGL (http://www.mricro.com, version v1.0.20180623).

The contrast Deviant > Standard, i.e. the contrast of triplets with low-probability endings (“Deviant”) and with high-probability endings (“Standard”), showed activations in the auditory cortex (STG) bilaterally, the orbitofrontal cortex (OFC) of the deep frontal operculum / the right anterior superior apex of the insula, the anterior portion of the middle cingulate gyrus (MCC) including area a24b, the rostral cingulate zone (RCZ), the pre-supplementary motor area (pre-SMA), the left precuneus, and the left putamen (see Fig. 2 and Table 1; results were corrected for multiple comparisons with voxel-wise control of the FDR at a threshold of *p* < 0.05). In the left hemisphere, activation was observed along the STG, the precuneus, and the putamen. The ROI analysis performed with the pars opercularis mask did not reveal any significant activation within this region (*p* = 0.43).

A second analysis was conducted to investigate changes in the activity pattern for deviance detection throughout the 6 blocks of the acquisition phase inside the scanner. By this, we sought to investigate whether the activity reflecting the deviance detection was influenced by the amount of time the participants were exposed to the stimuli. Expected differences would reflect the underlying learning process. This analysis did not yield any significant activations, indicating that there was no measurable differential response to deviances throughout the experiment.

Furthermore, a third analysis was conducted to investigate differences in activation patterns for the deviance detection between “good” and “bad” learners, based on their score in the familiarity test outside the scanner (see “Methods”). This analysis was performed as an independent samples t-test (using both SPM and LISA). The analysis did not reveal any significant difference in the activation associated with deviance detection in any of the nine ROIs listed in Table 1, between “good” and “bad” learners.

**Table 1:** Significant clusters activated more strongly during deviance detection.

Anatomical region	Hemisphere	MNI-coordinates			t-value	Cluster size
		x	y	z		
**Deviant > Standard**						
Superior and middle temporal gyrus and insula	L	− 56.50	0.81	− 4.51	0.99	147
(BA 22, BA 42, BA 21, BA 38)						
Superior and middle temporal gyrus and insula	R	56.81	7.48	− 8.40	1	367
(BA 22, BA 21, BA 42, BA 38)						
Putamen	L	− 16.50	10.81	− 8.40	0.97	10
Anterior superior apex of the insula and	R	33	31	3.27	0.98	31
orbitofrontal cortex of the deep frontal operculum						
Anterior portion of the middle cingulate gyrus	R	6.82	37.48	42.21	0.98	119
including area a24b’, anterior rostral cingulate zone						
and pre-supplementary motor area						
Premotor cortex	R	40.15	10.81	30.53	0.98	95
Posterior cingulate gyrus (BA 23)	R	3.49	− 32.51	30.53	0.98	12
Precuneus	L	− 3.17	− 79.17	46.10	0.98	35
Frontal lobe (BA 8)	L	− 3.17	27.48	50.00	0.97	7

Low-probability ending triplets contrasted to high-probability ending triplets, i.e., Deviant > Standard. The table shows the results that survived the correction for multiple comparisons (p < 0.05).

## Discussion

Our data reveal that processing of auditory irregularities, engages mainly the superior and middle temporal gyrus, the insula, the left putamen, the right frontal operculum, the posterior and middle cingulate gyrus, the premotor cortex, the precuneus and frontal lobe. This finding supports our hypothesis that auditory deviance detection within a statistical learning paradigm activates areas associated with the violation of local dependencies, as reported in previous artificial grammar learning studies. Contrary to our predictions based on literature, our neuroimaging results did not generate evidence regarding the time-course of statistical learning effects occurring during the scanning session, nor any significant differences between “good” and “bad” learners.

The extended activation in the left and right STG is in line with previous MMN studies^39–46^, and statistical learning studies^29,30,32^ where a similar activation pattern was observed along the temporal plane. As suggested in earlier statistical learning studies^30,33^, the STG supports the extraction of the statistical regularities based on the transitional probabilities of the stimuli and presumably, a similar process occurs also in the MMN oddball paradigm. Taken together, these results indicate the important role of the STG during the processing of structured stimuli, regardless of whether these are presented within an oddball MMN or a statistical learning paradigm. It is likely that the computation of stimulus statistics, namely the frequency of deviants’ occurrence, is a common process in both paradigms. However, in the case of statistical learning, the statistical cues are more sophisticated and thus more demanding in terms of processing effort and acquisition time, which presumably also explains the lack of activation in the primary auditory cortex (BA 41) in the current study.

In the current paradigm, deviance detection required listeners to implicitly learn the underlying structural properties of the stimuli, and not merely their acoustical properties. Contrary to a classical oddball paradigm where a change in the stimuli is instantly reflected in auditory memory responses, in the current paradigm a longer exposure time is required to learn the transitional probabilities. On this basis, detection of a statistical deviant engages a different cognitive mechanism than classical oddball stimuli^47–49^. Furthermore, a recent statistical learning study using EEG reported that brain responses elicited by statistical deviants had a more frontal scalp distribution than those elicited by location deviants^48^. This is in line with our findings showing stronger activations over the pre-SMA and premotor cortex compared to results from classical auditory oddball-studies. We suggest that the activations observed beyond the STG, namely in the right hemisphere cluster encompassing the OFC of the deep frontal operculum and the anterior superior apex of the insula, supported the establishment of the underlying structural relations (i.e., local dependencies) in the stimuli. This finding is reminiscent of findings from artificial grammar learning studies, showing that the processing of local dependencies does not necessitate Broca’s area but rather more posterior-medial areas of the frontal operculum, also referred to as the deep frontal operculum^20,50^. Furthermore, the deep frontal operculum and the premotor cortex may be regarded as being involved in the extraction and prediction of sequential auditory information. The role of premotor cortex goes beyond motor performance and is implicated in anticipatory and predictive processes (for a review see Ref.^51^). Previous studies examining violation of more abstract stimuli structures occurring in music and language have underlined the involvement of the inferior frontolateral cortex (BA 44), along with the premotor cortex, during both the recognition of regular structure and the detection of syntactical irregularities^52–54^. Thus, in the current study, it is likely that the OFC served an analogous role to that of the inferior frontolateral cortex (BA 44) but for sequential structures. A similar interaction between OFC, insular cortex and primary motor cortex has been reported in response to deviant events requiring spatial attention^55^. Taken together, these findings possibly suggest that the underlying mechanism of saliency detection is independent of stimulus modality.

In accordance with the predictive coding theory^56^, our findings indicate that predictive processes engaged during statistical learning and deviance detection, occur at different levels of the cortical hierarchy, including subcortical areas, sensory cortices and the prefrontal cortex. A recent study by Henin et al.,^57^ shows the hierarchical organisation of cortical circuits during statistical learning, where ascending brain structures are tracking higher-order information (i.e.: syllables vs words and pairs). In the current study, prediction errors (i.e., statistical deviants) are propagated bottom-up in the cortical hierarchy, whereas predictions are considered to be passed top-down, influencing the processing of new prediction errors at lower levels^58^. Thus, the OFC activity is suggested to reflect the updating of predictions regarding sequential regularities due to the processing of prediction errors. This finding is in line with previous research where the OFC has been shown to be sensitive to statistical parameters of the stimuli or breaches of expectations^59,60,60,61^. The OFC as one of the four emotional core systems^62^ is also implicated in music-evoked emotions^63^ such as surprise during expectancy violations^52^. Future research can further highlight how predictive coding mechanisms interact with emotions systems.

Another structure that has been suggested to encode error signals during predictive processes is the anterior cingulate cortex, of which the rostral parts show enhanced sensitivity to more abstract errors^64,65^. Several neuroimaging studies have illustrated the critical role of the anterior cingulate cortex during conflict monitoring and error processing^66,67^ (for a review see Ref.^68^). Moreover, statistical learning or artificial grammar learning studies have also reported activation of the anterior cingulate cortex or anterior parts of the medium cingulate gyrus, consistent with the notion that activation in this brain region reflects the cognitive demands of a task^16–18,29,31,33,46^. In light of this, deviance detection within our statistical learning paradigm is likely to reflect a state of error processing or conflict resolution which engaged the cingulate cortex, namely in the RCZ, the anterior portion of the MCC, and the pre-SMA. Nonetheless, the observed activation may also reflect a state of conflict associated with the cover task. Participants were asked to respond to the higher-pitched sound by pressing a button in the MRI-compatible handheld device. Thus, whenever a statistical deviance occurred, participants had to decide whether the deviant sound was a target sound or not, and to guide their behaviour accordingly (by not pressing the response button).

Taken together, our results point out that deviance detection, during ongoing learning of statistical regularities, is supported by a set of regions interacting with each other, where each region subserves a specific contribution to the overall process. It is very likely that in the current paradigm listeners were engaged in interdependent processes distributed over several brain structures and that the relative involvement of these brain structures was regulated in a dynamic way. This is the case when a situation requires constant cognitive evaluation that influences decisions for future actions. The constant interaction between processes is reflected by constant interaction between regions. For instance, the RCZ has been implicated in error processing and monitoring of behavioural outcome^69^ whereas the OFC has been implicated in the reward network and thus in the encoding of expected values^70^. Previous research has shown a constant interaction between RCZ and OFC in situations where participants have to adjust their behavioural outcome due to unexpected events, highlighting the close link between performance monitoring and decision making^69,71,72^. Similarly, Menon and Uddin^73^ noted the sensitivity of the insula to salient stimuli and subsequent interaction with the anterior cingulate to control the motor system. In this context, we suggest that our findings indicate an interaction between brain regions that support the overall process, mainly composed of the formation of predictions regarding regularities, the detection of salient events that may be either irregularities or target sounds, and finally the controlling of the behavioural response.

An additional finding of our study was the subcortical activation in the putamen (basal ganglia) during deviance detection. Although the basal ganglia are commonly connected to movement control, previous work has underlined their additional role in non-motor and language perception functions (for a review see Ref.^74^). Furthermore, activation of the basal ganglia has been reported during learning tasks; e.g. word segmentation in statistical learning^29,30^, processing of artificial grammar violations^21^, implicit category learning (for a review see Ref.^75^), action-sequence learning^76^, etc. However, our behavioral results did not provide evidence for ongoing learning inside the scanner and thus the putamen activation can be attributed to deviance detection, rather than learning. Yet, given that deviance detection tunes learning, it is highly likely that participants were still learning inside the scanner. Previous studies have shown that the BOLD response in the putamen correlates with the level of surprise elicited by the stimuli, thus this response likely reflected the prediction error^77–79^. Within the predictive coding framework, prediction errors, occurring also during deviance detection, play a fundamental role for learning. This issue needs to be specified in future studies.

## Limitations

Our neuroimaging results showed neither a learning effect during the fMRI session, nor a difference between “good” and “bad” learners. A post hoc power analysis using Neuropower resulted in a low estimate of statistical power, that is a possible indication of a small effect size. Specifically, based on our main SPM results, from the “good” vs “bad” learners second level contrast, the analysis conducted in Neuropower gave an uncorrected power of 0.98 and a familywise error rate (FWE) corrected power of only 0.27. It is also noteworthy, that due to the specifics of this study and its paradigm, deviant triplets had to be much less frequent than standard triplets. This unavoidably resulted in fewer data points for our experimental condition, which also impeded statistical power. Considering the aforementioned, our null result of the “good” and “bad” learners’ contrast, does not rule out the existence of important learning effects. Future studies could adopt the following measures to optimise their experimental design for the investigation of such learning effects: (i) performing more extensive training (e.g. with longer sessions and/or several sessions spread across several days with additional stimuli) to support all participants’ learning process; (ii) pre-screening in order to perform neuroimaging only with participants who show particularly good or particularly bad performance; (iii) using a larger sample for neuroimaging - based on our analysis, we would recommend N>40 per group; (iv) using more fMRI volume acquisitions per deviant stimulus - this could be accomplished by using simultaneous multislice acquisitions, to increase the fMRI time resolution by decreasing the TR to the range of 600–800 ms for each whole-brain volume acquisition (in that case an updated model of the hemodynamic response would also be preferable;^80^).

## Conclusion

In conclusion, our data reveal that the violation of local dependencies within a statistical learning paradigm engages a set of brain areas encompassing the STG, the insula, the deep frontal operculum, the RCZ, the MCC, the pre- SMA, and the putamen. The observed activation pattern is reminiscent of the left-hemispheric activation pattern observed during the violation of local dependencies based on speech sounds in artificial grammar learning experiments. The frontal contributions during statistical deviance detection corroborate our argument, that the irregularities occurring within the current paradigm are syntactic and necessitating predictive processes beyond the capabilities of auditory sensory memory. Based on the literature, we conclude that the observed activation pattern reflects the online adaptations required for predictive coding in the context of statistical learning.

## Methods

### Participants

Datasets from thirty-three participants were included in our analyses (16 females and 17 males; mean age = 24.97 years, *SD* = 5.60). All participants reported no hearing or language impairments, no history of neurological disease, nor musical training of more than 2 years besides regular school lessons. All participants received a compensation for their participation (200 NOK, approx. 20 EUR) at the end of the experiment.

### Ethics statement

The study was carried out in accordance with the guidelines of the Declaration of Helsinki, and was approved by the Regional Committee for Medical and Health Research Ethics for Western Norway with Reference Number: 2018/590. Participants provided written informed consent before the experiment.

### Stimuli

#### Sound triplets

To form the triplets we created six sounds. Each sound was a combination of a Shepard tone and a percussion sound. Shepard tones^81^ were employed to control for any possible effects of pitch along with any auditory grouping based on pitch. We generated six Shepard tones for six-note frequencies (F3: 174.61 Hz, G3: 196.00 Hz, A3: 220.00 Hz, B3: 246.94 Hz, C#4: 277.18 Hz, and D#4: 311.13 Hz); each tone resulted from the superposition of nine sinusoidal components spaced one octave apart. These six Shepard tones were combined with six percussive sounds (surdo, tambourine, agogo bells, hi-hat, castanet, and woodbloc) from an online library of sound samples by the Philarmonia Orchestra). All sounds were sampled at 44,100 Hz and normalised based on the RMS amplitude so that they matched in overall loudness. Each sound had a duration of 220 ms, including a fade-in ramp of 10 ms and a fade-out ramp of 20 ms. The interstimulus interval was 80 ms (thus, the inter-onset interval was 300 ms). The six sounds, corresponding to the letters A to F (see Fig. 1A), were combined into four triplets. Specifically, sounds A, B, C and D were combined in two (AB and CD) to form the “root” of the triplet—here “root” refers to the first two items of the triplet. Sounds E and F were used for the last position or item of the triplet. Thus, we obtained four unique triplets (Fig. 1A). Importantly, the arrangement of sounds (A to F) was permuted across participants to guarantee that possible acoustical differences between the sounds would not bias the results.

For the practice trials before the experiment, a second set of six sounds was created. These sounds were created similarly to the sounds of the main experiment, but the note frequencies of the Shepard tones differed (E3: 164.81 Hz, F#3: 184.99 Hz, G#3: 207.65 Hz, A#3: 233.08 Hz, C4: 261.62 Hz, and D4: 293.66 Hz) and the percussive samples were also different from those of the main experiment (woodblock, tambourine, agogo bells, castanet, hi-hat, and bass drum). Finally, a sound of much higher frequency was created (C#5: 554.37 Hz, not combined with a percussive sound), that was naturally audibly distinct from the rest of the stimulus set, to serve as a target sound for the cover task that participants had during practice trials and the experiment (see “Procedure”).

Triplets differed in respect to their frequency of occurrence in the experimental blocks. The “Standard” triplets comprised 90% of all presented triplets and featured endings with high transition probability (p = 0.9), whereas the “Deviant” triplets comprised 10% of all presented triplets and featured endings with low transition probability (p = 0.1). The current paradigm represents a 1st-order Markov model or bigram model with a strictly 2-local distribution (local dependencies^82^).

#### Triplet stream outside the scanner

400 triplets were pseudorandomly concatenated into pause-free streams or blocks of about 7 min duration each (see Fig. 1B). Triplets were presented in a pseudorandom order so that deviant triplets were separated by at least three standard triplets. No more than two consecutive and identical standard triplets were presented. Triplet roots (AB or CD) followed any of the two triplet endings (E or F) with a constant transitional probability (TP = 0.5). So, for example, ABE could be followed by either ABE, CDF, ABF, or CDE.

#### Triplet stream inside the scanner

For the in-scanner stream the transition probabilities were identical to the ones used outside the scanner (see Fig. 1B,C, for outside and inside the scanner respectively). The only modification of the in-scanner triplet stream was to account for the delay of the BOLD signal. Thus, every 27 standard triplets three consecutive deviant triplets were presented. Thus, trials of 30 triplets (27 standards followed by 3 consecutive deviant triplets) with a duration of 27 sec each were formed (see Fig. 1C). In each trial, standard triplets were presented in a pseudorandom order so that no more than two identical standard triplets were presented in direct succession.

### Procedure

#### Learning phase outside the scanner

To induce implicit statistical learning, participants underwent a learning (“familiarisation”) phase prior to the image acquisition. A room adjacent to the MRI scanner room was used during the learning phase of the experiment. Participants were asked to sit in a chair in front of a desk and listen to the sounds that would be presented to them via the headphones while watching a silent movie on the monitor in front of them. The experiment consisted of 3 blocks, each one comprised of an exposition phase of about 7 min followed by a behavioural test of about 2 min, resulting in a total duration of the learning phase of about 30 min.

The experiment started with a set of instructions presented on a computer display. Participants were not informed about the regularities in the arrangement of the stimuli, to ensure that any kind of learning throughout the experiment was implicit. At the same time, to ensure that participants were attentive to the stimuli, a cover task was used: The participants were asked to press the computer keyboard’s spacebar every time they heard the target sound (that consisted of a tone of the higher pitch without a percussive sound—see “Stimuli”). There were examples of the target sound in the instructions, followed by practice trials (lasting about 1 min) that contained a relatively high number of target sounds (8 target sounds). The practice trials were repeated if participants did not detect at least 80% of the target sounds or had a large number of false alarms (more than 3 false alarms).

#### Familiarity test and confidence rating outside the scanner

At the end of each block, an automated behavioural test assessed whether participants could distinguish (1) standard triplets from deviant triplets and (2) standard triplets from “non-triplets”, i.e., triplets that did not occur during the exposition phase (such as EFD, BDA, CFB, and ACE). Each test had twelve trials where participants were presented with twelve different triplet combinations (ABE vs. ABF, ABE vs. CDE, ABE vs. EFD, ABE vs. BDA, ABE vs. CFB, ABE vs. ACE, CDF vs. ABF, CDF vs. CDE, CDF vs. EFD, CDF vs. BDA, CDF vs. CFB, and CDF vs. ACE). In total, participants had to respond in thirty-six trials throughout the learning phase. There was a pause of 800 ms between the triplets. Participants were asked to choose which sequence sounded more familiar or caused them less of a surprise, using a 2-alternative forced choice test (pressing either “1” or “2” on the keyboard, to select either the first or the second sequence). Afterwards, they rated their level of confidence about their choice of sequence (by selecting a number on a scale that ranged from “1”—absolutely unsure, could have thrown a coin–to “5”—absolutely certain). Consecutive trials did not use the same triplet root and the presentation of triplet types was counterbalanced.

#### Acquisition and learning phase inside the scanner

After the learning phase outside the scanner, participants entered the scanning phase of the experiment during which learning was expected to continue. As mentioned earlier, the scope of the study was to induce implicit statistical learning both outside and inside the scanner and the only reason for modifying the triplet stream, for the period inside the scanner, was to account for the delay of the BOLD signal. Scanning comprised of a single run of approximately 31 min in duration (see Fig. 1C). In total, 6 blocks of 4.5 min duration each, interleaved with resting periods of 30 sec were presented. Within each block, 10 trials of 27 sec duration each were concatenated. In each trial, 27 consecutive standard triplets were presented followed by 3 deviant triplets. During the entire scanning session, a silent movie was projected on a screen located at the back of the scanner, which the participants could watch through a mirror display. To ensure that participants were attentive to the stimuli, the same cover task as that of the exposition phase was used: The participants were asked to press the button in the MRI-compatible handheld device with their index finger every time they heard the (higher-pitched) target sound. Participants were asked to lie still throughout the duration of the experiment to minimize noise. Auditory stimuli were presented via the MRI-compatible headphones and participants were provided with earplugs to alleviate any disturbances from the scanner noise.

### Image acquisition

The experiment was carried out using a 3T scanner (Siemens Prisma, Erlangen) and a 20-channel head coil. An anatomical reference T1-weighted ($T1_{w}$) image was acquired prior to the functional session, with voxel resolution = 1 $\times$ 1 $\times$ 1 mm$^{3}$, FOV = [220 220 144.08]. At the end of the anatomical scanning participants were reminded the instructions of the task. Functional T2-weighted images were acquired using a gradient-echo EPI sequence with voxel resolution = 3.3 $\times$ 3.3 $\times$ 3.3 mm$^{3}$, interslice gap = 0.594 mm and repetition time (TR) set at 2000 ms. In total 1074 volumes were acquired. The acquisition plane was tilted 30$^{\circ }$ from the AC-PC plane to decrease signal dropout in the orbitofrontal cortex^83^.

### Preprocessing

All preprocessing steps were performed using the fMRIPrep preprocessing pipeline^84^ apart from smoothing which was implemented in SPM12.

#### Anatomical data preprocessing

The T1-weighted (T1w) image was corrected for non-uniformity intensity using *N4BiasFieldCorrection*^85^, distributed with ANTs 2.2.0^86^, and used as T1w-reference throughout the workflow. The T1w-reference was then skull-stripped with a *Nipype* implementation of the *antsBrainExtraction.sh* workflow (from ANTs), using OASIS30ANTs as the target template. Brain tissue segmentation of cerebrospinal fluid (CSF), white-matter (WM) and gray-matter (GM) was performed on the brain-extracted T1w using *fast* (FSL 5.0.9^87^). Brain surfaces were reconstructed using *recon-all* (FreeSurfer 6.0.1^88^), and the brain mask estimated previously was refined with a custom variation of the method to reconcile the ANTs-derived and FreeSurfer-derived segmentations of the cortical gray-matter of Mindboggle^89^. Volume-based spatial normalization to the MNI standard space (MNI152NLin2009cAsym) was performed through nonlinear registration with *antsRegistration* (ANTs 2.2.0), using brain-extracted versions of both the T1w reference and the T1w template. The following template was selected for spatial normalization: *ICBM 152 Nonlinear Asymmetrical template version 2009c*^90^ (TemplateFlow ID:MNI152NLin2009cAsym).

#### Functional data preprocessing

For each BOLD run (1 per subject), the following preprocessing was performed. First, a reference volume and its skull-stripped version were generated using a custom methodology of *fMRIPrep*. The BOLD reference was then co-registered to the T1w reference using *bbregister* (FreeSurfer) which implements boundary-based registration^91^. Co-registration was configured with nine degrees of freedom to account for distortions remaining in the BOLD reference. Head-motion parameters with respect to the BOLD reference (transformation matrices, and six corresponding rotation and translation parameters) are estimated before any spatiotemporal filtering using *mcflirt* (FSL 5.0.9^92^). BOLD runs were slice-time corrected using *3dTshift* from AFNI 20160207^93^. The BOLD time-series, were resampled to surfaces on the *fsaverage5* spaces. The BOLD time-series (including slice-timing correction when applied) were resampled onto their original, native space by applying a single, composite transform to correct for head-motion and susceptibility distortions. These resampled BOLD time-series will be referred to as preprocessed BOLD in original space, or just preprocessed BOLD. The BOLD time-series were resampled into standard space, generating a *preprocessed BOLD run in [‘MNI152NLin2009cAsym’] space*. Several confounding time-series were calculated based on the *preprocessed BOLD*: framewise displacement (FD), DVARS and three region-wise global signals. FD and DVARS are calculated for each functional run, both using their implementations in *Nipype* (following the definitions by^94^). Three signals are extracted within CSF, WM, and whole-brain masks. Additionally, a set of physiological regressors were extracted to allow for component-based noise correction (*CompCor*^95^). Principal components were estimated after high-pass filtering the *preprocessed BOLD* time-series (using a discrete cosine filter with 128s cut-off) for the two *CompCor* variants: temporal (tCompCor) and anatomical (aCompCor). tCompCor components were then calculated from the top 5% variable voxels within a mask covering the subcortical regions. This subcortical mask was obtained by heavily eroding the brain mask, which ensures it does not include cortical GM regions. For aCompCor, components were calculated within the intersection of the aforementioned mask and the union of CSF and WM masks calculated in T1w space, after their projection to the native space of each functional run (using the inverse BOLD-to-T1w transformation). Components were also calculated separately within the WM and CSF masks. For each CompCor decomposition, the *k* components with the largest singular values were retained (so that the retained components’ time series are sufficient to explain 50% of variance across the nuisance mask - CSF, WM, combined, or temporal). The remaining components were dropped from consideration. The head-motion estimates calculated in the correction step were also placed within the corresponding confounds file. The confound time series derived from head motion estimates and global signals were expanded with the inclusion of temporal derivatives and quadratic terms for each^96^. Frames that exceeded a threshold of 0.5 mm FD or 1.5 standardised DVARS were annotated as motion outliers. All resamplings were performed with a *single interpolation step* comprised of all the pertinent transformations (i.e., head-motion transform matrices, susceptibility distortion correction when available, and co-registrations to anatomical and output spaces). Gridded (volumetric) resamplings were performed using *antsApplyTransforms* (ANTs), configured with Lanczos interpolation to minimize the smoothing effects of other kernels^97^. Non-gridded (surface) resamplings were performed using *mri_vol2surf* (FreeSurfer).

### Data analysis

#### Behavioural data analysis

Statistical analyses of the behavioural data comprised of the participants’ responses to the familiarity test and the cover task. Participants were asked to execute the same cover task outside and inside the scanner. The analyses were conducted using SPSS 25 (IBM Corp., Armonk, NY, USA). Responses were classified as correct when participants had correctly selected the sequence that represented a standard triplet (standard triplets had been played more frequently during the exposition phase). The mean percentage of correct responses was calculated for each participant and subsequently compared against chance level (0.5) using an independent samples t-test, $\alpha$ = 0.05.

#### 1st level data modeling

Using SPM12 we defined a general linear model (GLM) for the 1st-level of statistical inference. A high-pass filter with a cutoff frequency of 1/128 Hz was applied to remove low-frequency noise. An explicit binary mask, based on all subjects’ normalized gray matter images, was used. The 1st-level GLM included 26 regressors comprising of (a) two regressors for CSF and WM and (b) 24 regressors comprising the Volterra expansion of the realignment parameters. We performed 3 analytical investigations, corresponding to the 3 hypotheses of the study (see Introduction). For each subject we computed a contrast image between deviant and standard triplets, using an event-related design. That is, within each trial, the sequence of standard triplets was specified as one event of the standard condition and the sequence of the three final deviant triplets was specified as an event of the deviant condition. The condition of each event, along with its duration and its onset time in relation the beginning of the fMRI session, were specified for first level modelling in SPM12. A contrast vector was used to specify their comparison, resulting in the contrast images that were carried over to the second level of statistical modelling.

In relation to hypothesis (2), to model the evolution of deviance detection across the course of the experiment, an additional regressor was used, expressing the ‘Time x Condition’ interaction, that was constructed based on the onsets of the standard and deviant mini-blocks.

#### 2nd level data modeling

To control for Type I error the activation map of the second level contrast ‘Deviant > Standard’, as generated by SPM, was subjected to a probabilistic Threshold-free Cluster Enhancement (pTFCE,^98^) which yielded an effect at *p* = 0.05 corrected. In accordance with the three hypotheses, three analyses were conducted, respectively.

The first analysis aimed to investigate the brain responses regarding the detection of statistical deviance (‘deviance detection’). To assess whether the activations differed between the two conditions (Deviant and Standard) we conducted a group-level analysis (one-sample t-test). The analysis was performed using the Local Indicators of Spatial Association (LISA) tool^99^ which gives the advantage of a more sensitive analysis and ensures that even small activations are detected. LISA is a non-parametric and threshold-free framework that incorporates spatial context and thus preserves spatial precision without loss of statistical power. Within LISA, multiple comparison correction is achieved by controlling the false discovery rate (FDR) and thus there is no option for family-wise error correction. LISA takes into account topological features of the activation by applying a spatial filter to the z-map before the voxel-wise control of FDR. Control of FDR uses a Bayesian two-component mixture model and subsequent FDR scores for every voxel are estimated after 5000 random permutations. The individual contrast maps (as generated by SPM for the contrast Deviant > Standard) were subjected to a one-sample t-test using LISA.

An additional aim of the first analysis was to examine for a possible activation of the the pars opercularis of the IFG (BA 44i). For this purpose, we used FSLeyes (*McCarthy*, 2021; http://doi.org/10.5281/zenodo.4704476) and the Harvard-Oxford Cortical Structural Atlas to create a thresholded ROI mask for the pars opercularis. Subsequently, the ROI analysis was conducted using the SPM toolbox MARSeille Boîte À Région d’Intérêt (MarsBar).

The second analysis examined how deviance detection evolved over the course of the experiment. Finally, the third analysis examined whether there was any quantitative difference between “good” and “bad” learners within the regions of interest for the deviance detection.

For the third analysis, we removed three subjects; two were missing data on the familiarity test and one other was confused and performed too many false alarms (132 false alarms). The 30 subjects were divided into two groups of “good” (14 subjects) and “bad” (16 subjects) learners based on the median value of their scores in the pre-fMRI learning task. We restricted the comparison within nine anatomical ROIs corresponding to the nine clusters that showed significant activation during the processing of the deviant vs standard triplets (see Table 1). We used the SPM toolbox MarsBaR to estimate a mean value for each participant in each of the nine clusters, from the contrast ‘Deviant > Standard’. Subsequently, these mean values were entered in nine two-sample t-tests using MATLAB^®^ to investigate possible differences between the two groups, during deviance detection in any of the nine ROIs.

The results were visualized using xjView toolbox (https://www.alivelearn.net/xjview) and Fig. 2 was created using MRIcroGL (http://www.mricro.com, version v1.0.20180623) (Supplementary information S1).

## Supplementary Information

Supplementary Information.

## Data Availability

Dataset URL in a public repository: https://www.kaggle.com/vtsogli/fmri-local-dependencies-statistical-learning.
