# Supplementary material for: Brain-correlates of processing local dependencies within a statistical learning paradigm
Source: Sci Rep. 2022 Sep 12;12:15296. doi: 10.1038/s41598-022-19203-7 (PMC9468168; doi:10.1038/s41598-022-19203-7)
Supplement: Supplementary file 1 — Supplementary Information. [file 41598_2022_19203_MOESM1_ESM.pdf]

Supplementary Material

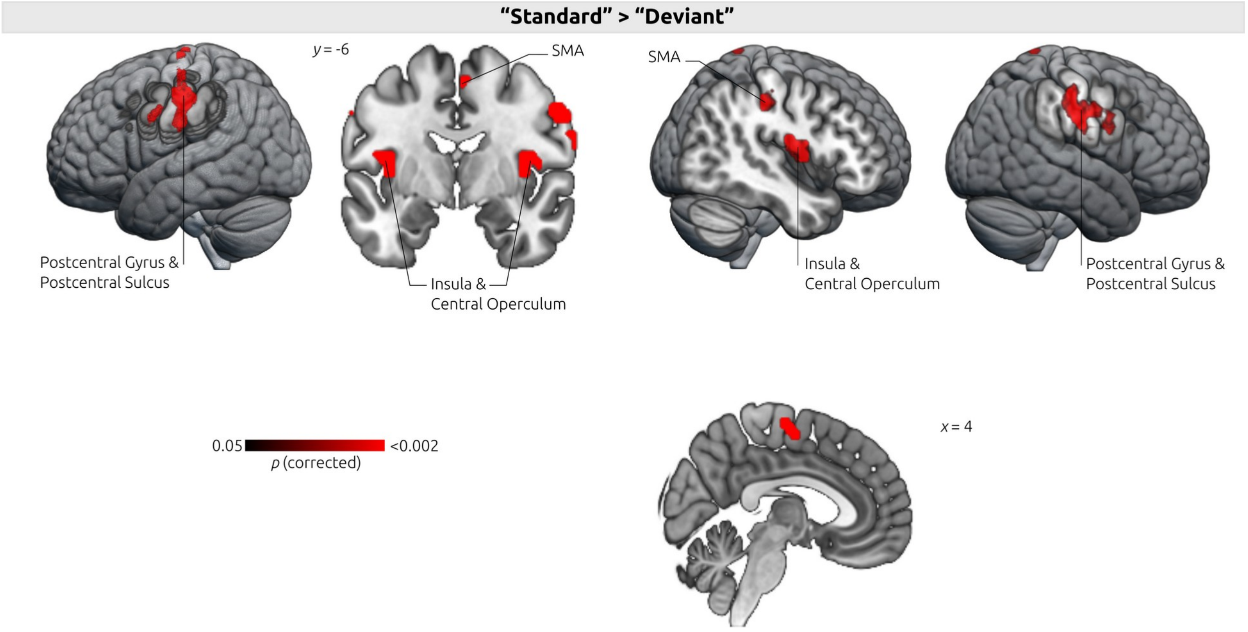

Figure 3: Brain activation pattern during "Standard" > "Deviant". Activation map thresholded at p < 0.05 (FDR). Default rendering was used in MRIcroGL and the opacity was adjusted to reveal activations recorded deeper in the brain. Supplementary Motor Area (SMA).

Anatomical region	Hemisphere	MNI-coordinates			t-value	Cluster Size
		x	y	z		
**Standard > Deviant**						
Insula	L	-36.51	-5.85	7.17	0.97	39
Insula	R	36.81	-5.84	7.16	0.98	62
Inferior parietal lobule	R	56.81	-15.84	34.42	0.98	202
Inferior parietal lobule	L	-56.50	-22.51	42.21	0.98	107
SMA	R	3.50	-9.18	57.80	0.97	24
Central sulcus	L	-19.84	-22.51	77.26	0.97	18
SPL	R	16.82	-45.84	77.27	0.96	5

Table 1. Significant clusters activated more strongly for deviance detection for the contrast (high-probability ending triplets contrasted to low-probability ending triplets, i.e., Standard > Deviant). The table shows the results of the cluster analysis of statistical parametric maps ($p < 0.05$, FDR corrected for multiple comparisons).
